# Supplementary material for: Spin-orbit torque switching of an antiferromagnetic metallic heterostructure
Source: Nat Commun. 2020 Nov 11;11:5715. doi: 10.1038/s41467-020-19511-4 (PMC7658218; doi:10.1038/s41467-020-19511-4)
Supplement: Supplementary file 1 — Supplementary Information [file 41467_2020_19511_MOESM1_ESM.pdf]

Spin-orbit torque switching of an antiferromagnetic metallic heterostructure

Samik DuttaGupta[1-3]*, A. Kurenkov[1-3], Oleg A. Tretiakov[4], G. Krishnaswamy[5], G. Sala[5], V. Krizakova[5], F. Maccherozzi[6], S. S. Dhesi[6], P. Gambardella[5], H. Ohno[1-3,7,8] and S. Fukami[1-3,7,8]

[1]*Center for Science and Innovation in Spintronics, Tohoku University, 2-1-1 Katahira, Aoba-ku, Sendai 980-8577, Japan*
[2]*Center for Spintronics Research Network, Tohoku University, 2-1-1 Katahira, Aoba-ku, Sendai 980-8577, Japan*
[3]*Laboratory for Nanoelectronics and Spintronics, Research Institute of Electrical Communication, Tohoku University, 2-1-1 Katahira, Aoba-ku, Sendai 980-8577, Japan*
[4] *School of Physics, The University of New South Wales, Sydney 2052, Australia*
[5]*Laboratory for Magnetism and Interface Physics, Department of Materials, ETH Zurich, 8093 Zurich, Switzerland*
[6]*Diamond Light Source, Chilton, Didcot, Oxfordshire, OX11 0DE, United Kingdom*
[7]*Center for Innovative Integrated Electronic Systems, Tohoku University, 468-1 Aramaki Aza Aoba, Aoba-ku, Sendai 980-0845 Japan*
[8]*WPI Advanced Institute for Materials Research, Tohoku University, 2-1-1 Katahira, Aoba-ku, Sendai 980-8577, Japan*
Corresponding author: sdg@riec.tohoku.ac.jp

SUPPLEMENTARY INFORMATION

S1. X-ray diffraction of PtMn thin films

S2. Current-induced switching of PtMn(30)/Pt structures

S3. Comparison of electrical switching characteristics between PtMn(10)/Pt and Pt(5)/Ru structures

S4. Current-polarity dependence of switching in PtMn(t_{PtMn})/Pt structures

S5. Current-induced manipulation of PtMn(10 and 30)/Ru structures

S6. Current-induced switching of PtMn(10)/Pt structure utilized for X-Ray Magnetic Linear Dichroism (XMLD) measurements

S7. Estimation of Dzyaloshinskii-Moriya constant (D) for PtMn/Pt structures

S8. Estimation of critical Dzyaloshinskii-Moriya constant (D_C) for inhomogeneous antiferromagnetic ground state in AFM/HM structures

S9. Estimation of antiferromagnetic domain wall (DW) width

S10. Calibration of temperature rise due to current pulse injection

S11. Optical micrograph of PtMn/Ru structures after current pulse application

S1. X-ray diffraction (XRD) of PtMn thin films

The phase diagram for bulk PtMn around Mn-composition 40-65% shows three possible, stable crystallographic phases: a high-temperature liquid phase, chemically ordered B2 phase within the temperature range 1100-1700 K, and a chemically ordered antiferromagnetic $L1_0$ phase below 1100 K [S1]. In thin film form, it is widely known that sputter-grown as-deposited PtMn is paramagnetic with a disordered, face-centered-cubic (fcc) structure [S2, S3]. A post deposition annealing is required to transform it to antiferromagnetic $L1_0$-ordered phase with a face-centered-tetragonal (fct) structure, manifested as a gradual shift the XRD peaks associated with a change in lattice spacings [S2]. Note that the effect of post-deposition annealing is only to provide the necessary activation energy required for atomic rearrangement. To clarify the crystalline orientation of the antiferromagnet (AFM) PtMn, we carry out out-of-plane (θ - 2θ) XRD measurements using Ta(3)/Pt(3)/MgO(2)/PtMn(10)/Ru(2) structures for both as-deposited and annealed states. Figure 1 shows the XRD scans for as-deposited films and films annealed at 300 °C for 2 hr in the presence of an in-plane magnetic field. Only reflections from fcc or fct-PtMn(111) and fcc-Pt(111) are observed, indicating that PtMn and Pt layers have (111) orientation of fcc (or $L1_0$-ordered fct for PtMn) phase. Clearly, the (111) planar spacings changes from 2θ= 39.80° ± 0.02° for the as-deposited to 2θ= 40.20° ± 0.02° for the annealed thin films. This shift of the (111) peak position is expected to originate from the phase transformation from disordered fcc to $L1_0$-ordered tetragonal structures. While we cannot exactly quantify the degree of phase transformation in our thin films, we would like to point out that the observed 2θ value of 40.20° ± 0.02° matches with the one expected after completion of volume phase transformation [S2].

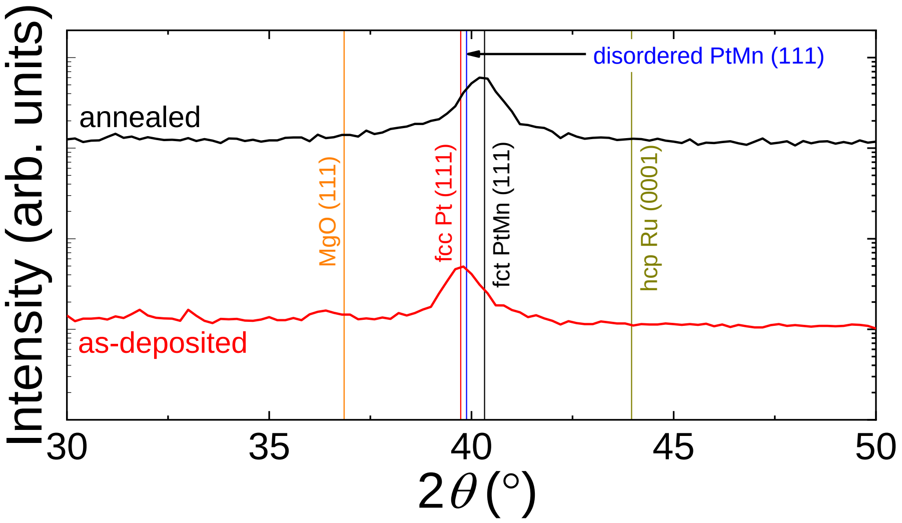

Figure S1: Out-of-plane X-ray diffraction (XRD) patterns for Ta(3)/Pt(3)/MgO(2)/PtMn(10)/Ru(2) heterostructures in as-deposited and annealed state, respectively. Annealing conditions are the same as mentioned in the main text. Vertical lines in the figure denotes the expected peak positions for (from left) MgO (111), fcc-Pt (111), disordered fcc-PtMn (111), fct-PtMn (111) and hcp-Ru (0001).

S2. Current-induced switching of PtMn(30)/Pt structures

In the main manuscript, we have demonstrated current-induced switching of antiferromagnetic Néel vector by spin-orbit torques (SOT) in PtMn(10)/Pt structures. To clarify the role of AFM in the current-induced switching of AFM/heavy-metal (HM) heterostructures, we have carried out electrical measurements on PtMn(t_{PtMn})/Pt structures for various PtMn thicknesses (t_{PtMn}). Figures S2 (a) and (b) show the experimental results of applied current-density and pulse width (τ_P) dependence of switching with an increase of t_{PtMn} up to ~ 30 nm in PtMn(30)/Pt structures of identical device dimensions as mentioned in the main text. Distinct reversible resistive modulation of the Hall resistance (R_{Hall}) under the application of orthogonal current pulse injection persists with an increase of t_{PtMn}. Similar to PtMn(10)/Pt, the degree of current-induced switching (ΔR_{Hall}) of the AFM increases with the increase of applied write current density in the Pt layer (J_{Pt}) and τ_P. The independence of the observed results with a variation of t_{PtMn} indicates the intrinsic nature of the observed behavior for PtMn(t_{PtMn})/Pt structures.

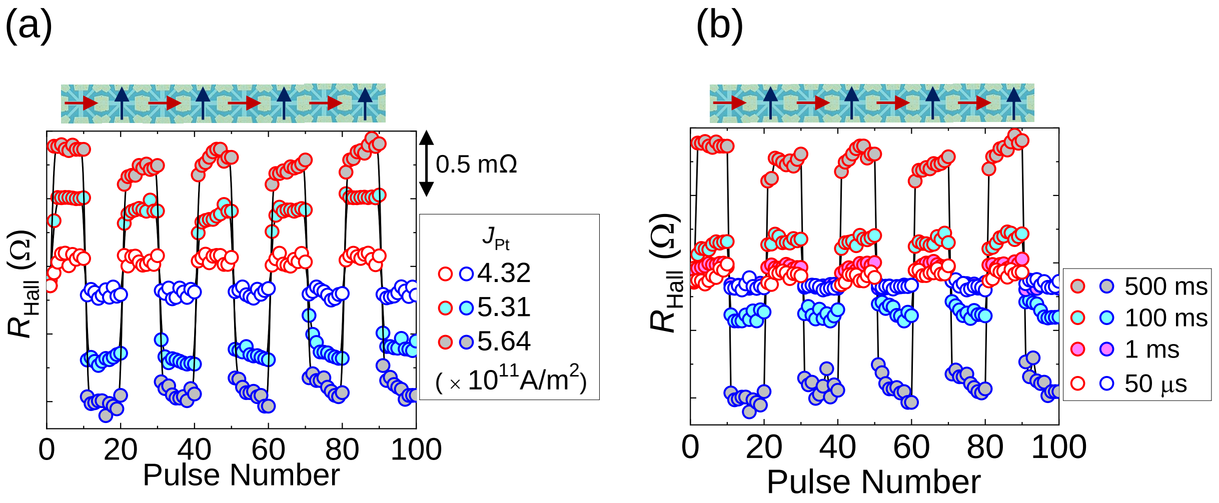

Figure S2: (a) Experimental results of current-induced switching of PtMn(30)/Pt structures under applied write current densities (J_{Pt}) = 4.32 ×10^{11} A/m^2 (J_{PtMn} = 4.96 × 10^{10} A/m^2), 5.31 × 10^{11} A/m^2 (J_{PtMn} = 5.95 × 10^{10} A/m^2) and 5.64 ×10^{11} A/m^2 (J_{PtMn} = 8.17 × 10^{10} A/m^2). (b) Pulse width dependence of Hall resistance (R_{Hall}) of the same structure under applied J_{Pt} = 5.64 ×10^{11} A/m^2. Schematic diagrams above fig. (b) indicate the direction of the applied write current pulses. Scale bar for the vertical axis is the same for both (a) and (b).

S3. Comparison of electrical switching characteristics between PtMn(10)/Pt and Pt(5) structures

Our experimental results on PtMn(10)/Pt and PtMn(10) structures demonstrate electrical writing of information in the metallic AFM by spin-orbit torques (SOTs). However, recent experimental results in AFM/HM structures have pointed towards thermoelectric origins of the observed voltage/resistance changes, not related to any changes of the antiferromagnetic Néel vector [S4]. To clarify the origin of the observed changes in Hall resistance (R_{Hall}), we compare our experimental results in sub./Ta(3)/Pt(2)/MgO(2)/PtMn(10)/Pt(5)/Ru(1) (PtMn(10)/Pt, hereafter) with similar experiments in sub./Ta(3)/Pt(5)/Ru(1) (Pt(5)/Ru, hereafter) structures. The deposited Pt(5)/Ru films were patterned into similar 8-terminal device structures (as in the main text). Five current pulses are sourced along the horizontal arm (A → B), followed by five pulses in the transverse direction (C → D). The polarities of $I_{1,2}$ are the same as the experimental configuration in the main text. Figures S3(a) and (b) shows the experimental results of current-induced switching in PtMn(10)/Pt and Pt(5)/Ru, respectively. Miniscule changes in R_{Hall} are observed for Pt(5)/Ru structure as compared to PtMn(10)/Pt for comparable magnitudes of applied write current densities in the Pt layer. This excludes the possibility of a dominant contribution of thermoelectric voltages to the observed R_{Hall} in PtMn/Pt structures.

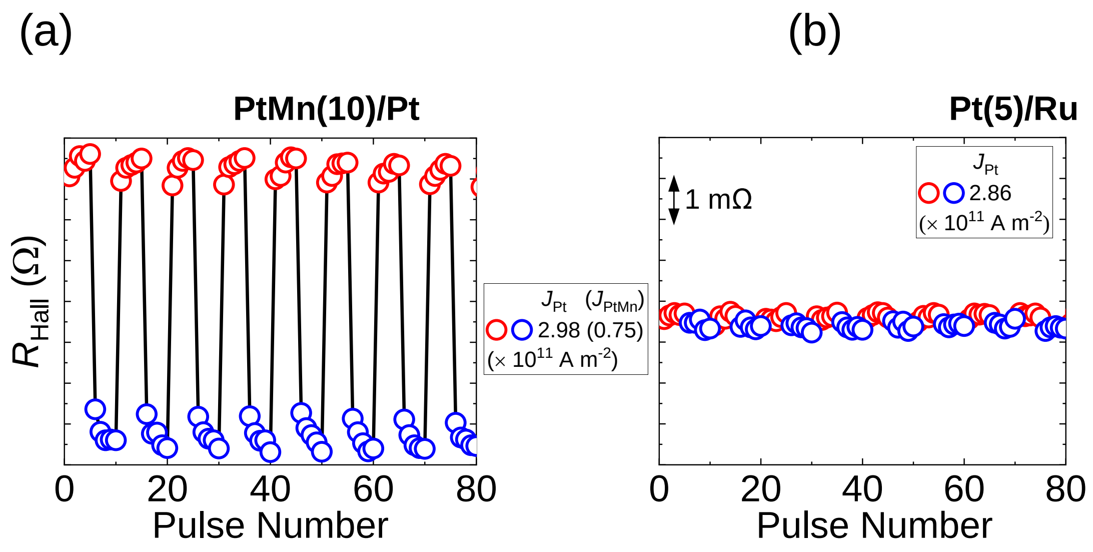

Figure S3: (a) Experimental results of current-induced switching of PtMn(10)/Pt structures under applied write current densities (J_{Pt}) = 2.98 $\times 10^{11}$ A/m^2 (J_{PtMn} = 7.51 $\times$ 10^{10} A/m^2). (b) Experimental results of similar measurements in Pt(5)/Ru structures under applied current densities (J_{Pt}) = 2.86 $\times 10^{11}$ A/m^2. Scale bar for the vertical axis is the same for both (a) and (b).

S4. Current-polarity dependence of switching in PtMn(t_{PtMn})/Pt structures

Our experimental results on PtMn/Pt and PtMn/Ru structures indicate a different origin of the current-induced switching characteristics, highlighting the different effects of the current between these structures. Here, we show a second distinct feature of the current-induced switching behavior in PtMn/Pt structures. Figure S4 (a) and (b) show the experimental results of current-induced switching experiments for various τ_P in PtMn(10)/Pt and PtMn(30)/Pt structures, respectively. The polarities of I_1 and I_2 are along D(B)→ C(A), as opposed to A(C)→ B(D) in the main text and supplementary section S1. A reversal of $I_{1,2}$ polarities results in a reversal of R_{Hall} for the PtMn/Pt structure. Interestingly, this bi-polar nature of current-induced switching is completely absent in PtMn structures without the HM layer (as will be shown in supplementary S5). While the origin of this behavior is still not well understood at present, we note that current-polarity dependent switching has been previously observed in CuMnAs, which was attributed to the current-induced motion of the antiferromagnetic domain walls (DWs) [S5]. A second possible origin for the observed behavior relates to the complicated roles played by uncompensated surface moments or possible non-collinear spin structures [S6, S7].

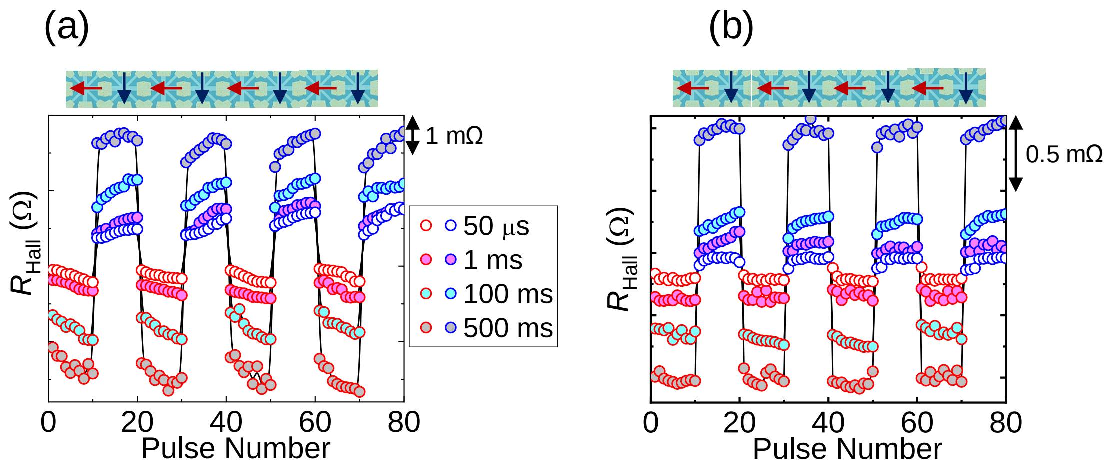

Figure S4: (a) Experimental results of current-induced manipulation of PtMn(10)/Pt structures under applied write current densities J_{Pt} = 3.27 ×10^{11} A/m^2 (J_{PtMn} = 8.17 × 10^{10} A/m^2) for pulse widths (τ_P) = 50 μs, 1 ms, 100 and 500 ms. Write current polarities are indicated in the schematics above the figures and opposite to that shown in the main text. (b) Pulse width dependence of current-induced switching of PtMn(30)/Pt structure under applied J_{Pt} = 5.64 ×10^{11} A/m^2 (J_{PtMn} = 8.17 × 10^{10} A/m^2). Schematic diagrams above fig. (b) indicate the direction of the applied write current pulses. Reversal of Hall resistance (R_{Hall}) is observed with the reversal of write current polarities irrespective of the thickness of the antiferromagnetic layer.

S5. Current-induced manipulation of PtMn(10 and 30)/Ru structures

The injection of current through the metallic AFM/HM heterostructure results in a finite current flow through both the AFM and NM layers. Different dynamics of magnetization can be induced owing to the current flow through the HM and the AFM layer [S8- S13]. Thus, understanding the current-induced manipulation results for AFM/HM structures also requires the quantification of current-induced effects on the AFM itself. Here, we experimentally evaluate the impact of current in sub./Ta(3)/Pt(2)/MgO(2)/PtMn(t_{PtMn})/Ru(1) (PtMn(t_{PtMn})/Ru, hereafter) structures. The device structure and the experimental configuration are identical to the one as mentioned in the main text. Write pulses ($I_{1,2}$) are injected along two orthogonal directions, and the transverse Hall resistance (R_{Hall}) is measured. Figure S5(a) and (b) show the experimental results of current-induced manipulation of PtMn(10)/Ru and PtMn(30)/Ru structures while S5(c) and (d) show the pulse width dependence of switching for these structures respectively. In contrast to the experimental results on AFM/HM structures, R_{Hall} shows a linear non-saturating increase (decrease) with the application of successive current pulses along I_1 (I_2). A change in write pulse amplitude results in an increase of R_{Hall} for both the structures, indicative of current-induced effects of the AFM. The different dynamics of the antiferromagnetic heterostructure under the application of current is indicative of different underlying dynamics of the antiferromagnetic Néel vector for AFM/HM and AFM heterostructures. Figure S5(c) and (d) show the τ_P dependence of R_{Hall} for PtMn(30)/Ru heterostructure under applied current density J_{PtMn} = 2.11 × 10^{11} A/m^2 with a change in the direction of the write pulses (→↑ and ←↓) as mentioned in the insets. As opposed to the experimental results on AFM/HM structures, the nature of R_{Hall} is insensitive to the direction of current in PtMn/Ru heterostructures. The present results highlight different underlying factors for antiferromagnetic Néel vector manipulation between PtMn/Pt and PtMn/Ru heterostructures. The underlying differences in the current-induced manipulation of these structures are discussed in the main text.

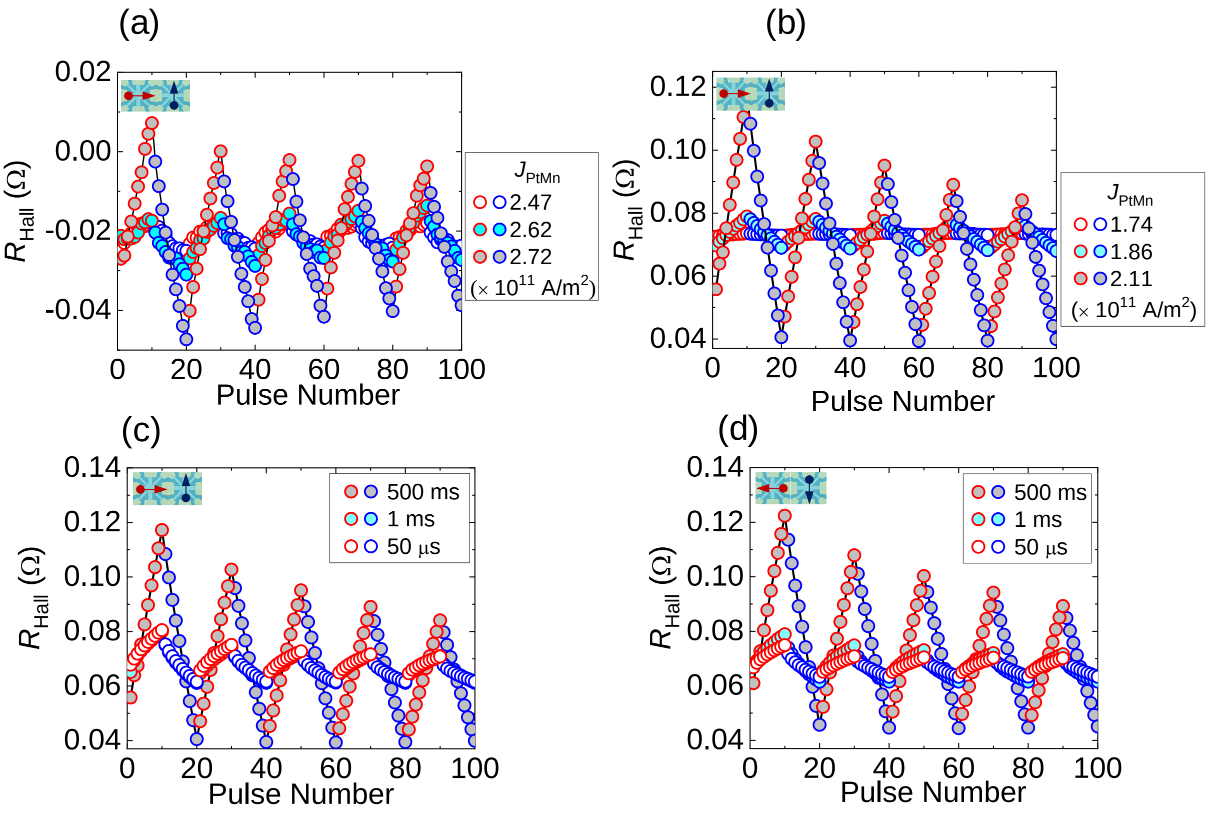

Figure S5: (a), (b) Experimental results of current-induced manipulation of PtMn(10)/Ru and PtMn(30)/Ru structures under applied write current pulses J_{PtMn} of 500 ms duration. Schematic micrographs above the figures indicate the direction of applied current pulses. A gradual triangular variation of the Hall resistance (R_{Hall}) is observed as opposed to the step-like behavior for PtMn/Pt structures. (c),(d) Pulse width dependence of Hall resistance (R_{Hall}) for PtMn(30)/Ru structure under applied write current J_{PtMn} = 2.11 $\times 10^{11}$ A/m^2 for opposite directions of the write current pulses. The schematic micrographs in the figure indicate the direction of the applied write current pulse.

S6. Current-induced switching of PtMn(10)/Pt structure utilized for X-Ray Magnetic Linear Dichroism (XMLD) measurements

Electrical measurements of current-induced switching were also carried out in the AFM/HM samples utilized for XMLD investigations. The device size and experimental configuration are identical to the one, as mentioned in the main text. Separate devices fabricated from the same wafer were utilized for XMLD measurements indicated in the main text. Figure S6 shows the experimental results of Hall resistance (R_{Hall}) under orthogonal current pulse injection at writing current densities of $J_{Pt} = 5.93 \times 10^{11}$ A/m^2 for applied pulse widths of 100 and 500 ms respectively. Similar bi-stable resistive states are obtained under the injection of current pulses, as shown in the main text. The present results confirm the reproducibility of the electrical measurements and enable a visualization of the antiferromagnetic dynamics under current-pulse injection.

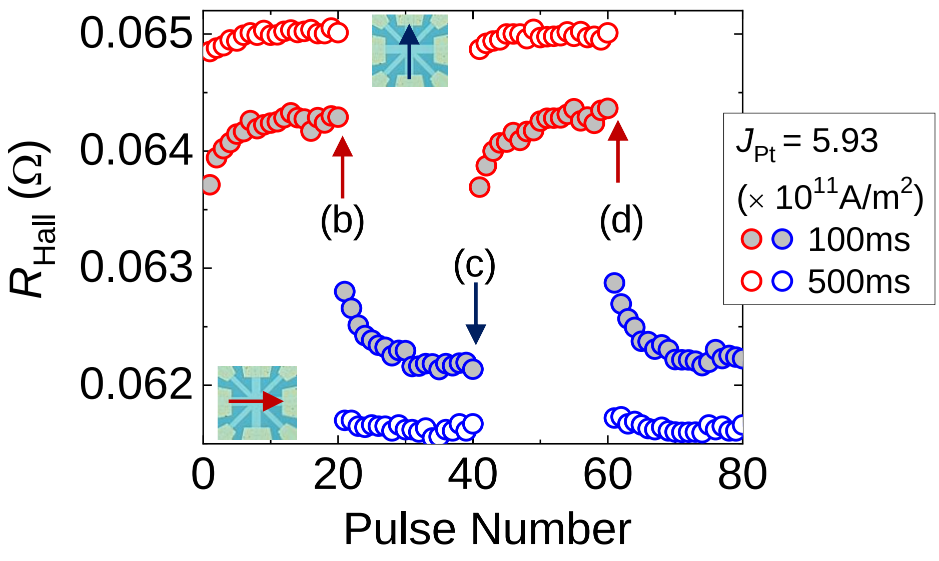

Figure S6: Experimental results of current-induced switching of Pt/PtMn(10) structures under orthogonal write pulse injection at current densities of J_{Pt} = 2.11 ×10^{11} A/m^2 for 100 and 500 ms pulse durations. Schematic micrographs shown inside the figure indicate the direction of the applied write pulse in a four-terminal geometry. Arrows in the figure indicate the position on the curve where the XMLD data is acquired and shown the main text (Fig.4 of the main text).

S7. Estimation of Dzyaloshinskii-Moriya constant (*D*) for PtMn/Pt structures

The clarification of Dzyaloshinskii-Moriya interaction (DMI) at AFM/HM interfaces and estimation of DMI constant (D) is expected to provide crucial insights concerning current-induced switching of AFM/HM structures. Thus, we estimate the range of D for various magnitudes of K_U, A_1 and A_2 using the following analytical relations as [S14]

$$D = \frac{16|K_U|\delta^2}{\pi\lambda} E\left(\frac{1}{\sqrt{C}}\right) K\left(\frac{1}{\sqrt{C}}\right) \quad (1)$$

where δ is the antiferromagnetic DW width, A_1 (> 0) corresponds to the inhomogeneous component of intra sublattice exchange constant, A_2 (< 0) is the inhomogeneous component of the inter-sublattice exchange constant, and K_U is the anisotropy constant, and λ corresponds to antiferromagnetic domain size. $E\left(\frac{1}{\sqrt{C}}\right)$ and $K\left(\frac{1}{\sqrt{C}}\right)$ are first and second-order complete elliptic integrals, where C is an integration constant. Using $\lambda \approx 100$ nm, from X-ray microscopy measurements (Fig. 4(l) of the main manuscript) and using the values of K_U, A_1, and A_2 from previous reports [S14-S17], we obtain the variation of D versus C (Figure S7). The range of C is constrained ($0 < C \leq 1$) within the two divergent limits of the elliptic integrals. As stated in the main manuscript, this estimate of D is close to a critical DMI constant (D_C), calculated with typical parameters reported for Mn-based metallic AFMs.

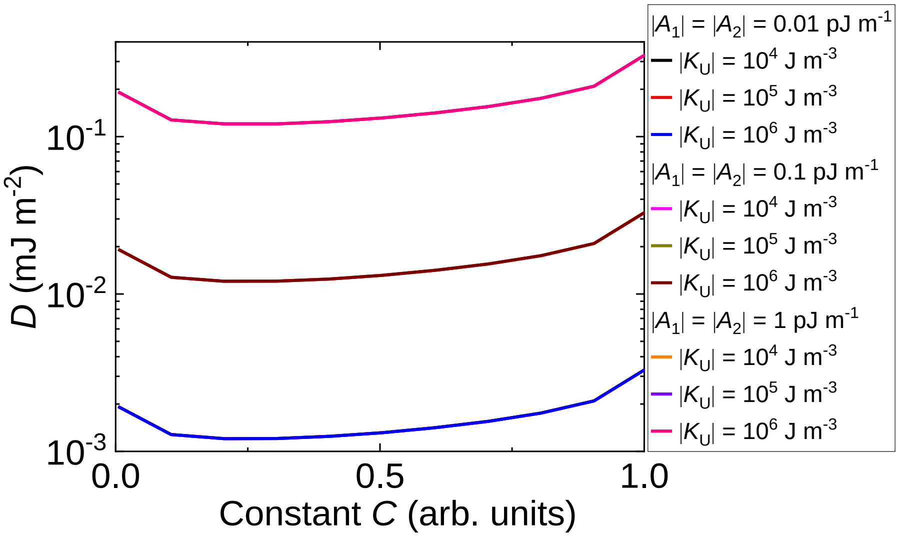

Figure S7: Estimation of DMI constant (D) versus integration constant (C), using the analytical formula. The range of C ($0 < C \leq 1$) is constrained within this limit owing to the diverging values of D for C =0, and C >1, respectively.

S8. Estimation of critical Dzyaloshinskii-Moriya constant (D_C) for multi-domain antiferromagnetic ground state in AFM/HM structures

For ferromagnet/HM multilayer systems, the presence of broken inversion symmetry results in interfacial chiral interactions (*e.g.*, DMI) which renders the stabilization of chiral spin textures (chiral Néel DWs or skyrmions) and shown to play an important role concerning SOT-induced magnetization switching. Owing to the ubiquitous presence of broken inversion symmetry, understanding and clarification of the role of DMI in AFM/HM structures are extremely important. Recent analytical and micromagnetic calculations have shown that the presence of DMI can stabilize a multi-domain inhomogeneous ground state in AFMs with easy-plane anisotropy component when the interfacial DMI constant (D) is comparable or larger than a critical value (D_C) [S14, S15]. This critical value of D_C is given by

$$D_C = \frac{2}{\pi}\sqrt{2(2A_1 - A_2)|K_U|} \quad (2)$$

where, A_1 (> 0) corresponds to the inhomogeneous component of intra sublattice exchange constant, A_2 (< 0) is the inhomogeneous component of inter-lattice exchange constant, and K_U is the anisotropy constant. Table T1 shows the estimate of D_C for A_1, A_2, and K_U within the range of typical values characteristic to Mn-based metallic AFMs. Interestingly, the estimated values of D_C are comparable to an estimation of D for PtMn/Pt structures. Note that D_C is also comparable to that for Pt/FM interfaces [S18]. Our results show a crucial role played by chiral interactions towards understanding of current-induced switching in AFM/HM structures.

A_1 (pJ m^{-1})	A_{12} (pJ m^{-1})	K_U (J m^{-3})	D_C (mJ m^{-2})	A_1 (pJ m^{-1})	A_{12} (pJ m^{-1})	D_C (mJ m^{-2})
0.01	-0.01	1×10^4	0.01	0.1	-0.1	0.05
0.01	-0.01	1×10^5	0.05	0.1	-0.1	0.15
0.01	-0.01	1×10^6	0.15	0.1	-0.1	0.49
1	-1	1×10^4	0.15	10	-10	0.49
1	-1	1×10^5	0.49	10	-10	1.56
1	-1	1×10^6	1.56	10	-10	4.93

Table T1: Estimate of critical Dzyaloshinskii-Moriya constant (D_C) for various magnitudes of anisotropy constant (K_U) and inhomogeneous component of intra sub lattice (A_1) and inter sublattice (A_2) exchange constants.

S9. Estimation of antiferromagnetic domain wall (DW) width

The static DW width for a two sublattice collinear AFM can be analytically expressed as [S14, S15]

$$\delta = \sqrt{\frac{2A_1 - A_2}{2|K_U|}} \quad (3)$$

where, A_1 (> 0) corresponds to the inhomogeneous component of intra sublattice exchange constant, A_2 (< 0) is the inhomogeneous component of inter-lattice exchange constant, and K_U is the anisotropy constant. Note that the analytical expression for δ is dependent only on the inhomogeneous components of exchange and magnitude of anisotropy constants, while is independent of the homogeneous component of exchange and the nature of the magnetic anisotropy (uniaxial or easy plane). Figures S8(a)-(c) shows the variation of δ versus A_1, A_2, and K_U, where the range of the variable parameters were chosen as in previous studies [S14-S17]. The obtained values of δ are smaller than the crystallite size ~ 10 nm (from X-ray diffraction), and the switchable antiferromagnetic domain size of ~ 100-200 nm (from XMLD measurements), indicating the feasibility of the antiferromagnetic spin texture-dominated reversal of antiferromagnetic Néel order parameter.

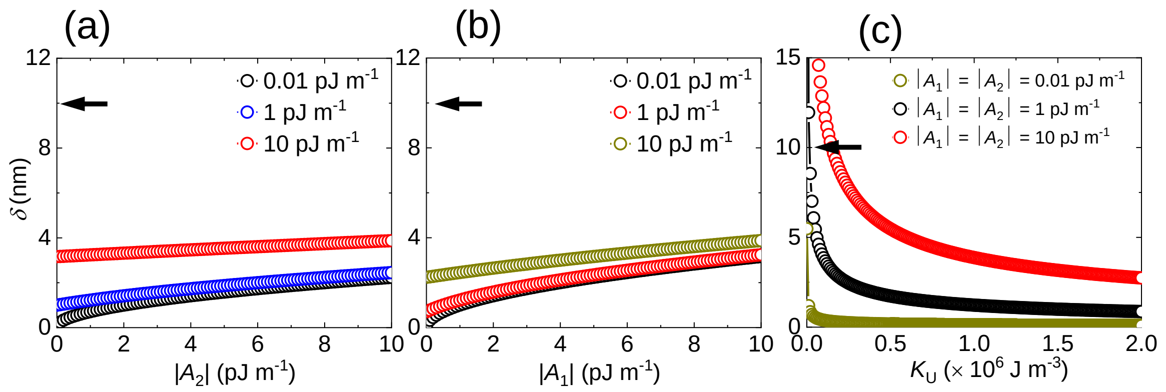

Figure S8: (a) AFM DW width (δ) versus $|A_2|$ for $|A_1|$ = 0.01, 1 and 10 pJ m^{-1} at $K_U = 10^6$ J m^{-3}. (b) variation of δ versus $|A_1|$ for $|A_2|$ = 0.01, 1 and 10 pJ m^{-1} at $K_U = 10^6$ J m^{-3}. (c) variation of δ versus K_U for different values of A_1 and A_2 spanning over four orders of magnitude. Black arrows in the figure indicate the mean crystallite size obtained from X-ray diffraction measurements.

S10. Calibration of temperature rise due to current pulse injection

The injection of current in PtMn/Pt and PtMn/Ru structures leads to an increase in temperature due to Joule heating. This temperature rise has been shown to result in significant effects on the antiferromagnetic Neel vector in other systems [S10, S11]. Thus, it is imperative to quantify the temperature rise in our PtMn/Pt and PtMn/Ru structures. The temperature rise is calibrated by measuring resistance of PtMn/Pt and PtMn/Ru for various applied dc current and converting the change of resistance into the change in temperature. Figure S9(a), (c) shows that the resistivity of the device increases with temperature, as expected for metallic systems. The temperature coefficient of the resistance is obtained from a fit of the temperature dependence of the resistivity measured between 50 and 300 K. The resistivity of the device increases as a function of current density in the PtMn layer J_{PtMn}. The obtained dependence can be fitted with a quadratic function CJ^2 (not shown), from which the coefficient (C) of resistivity as a function of J is obtained. From these results, the temperature rise is calculated, which is shown as a function of J_{PtMn} in Fig. S9(b). Owing to the larger current flow through the PtMn layer in PtMn(t_{PtMn})/Ru as compared to PtMn(t_{PtMn})/Pt structures, a larger temperature rise in PtMn/Ru structure occurs. The maximum temperature rise in PtMn/Ru at the highest applied current density is larger by more than a factor of 2 compared to PtMn/Pt. These results evidence the non-negligible role played by the temperature rise with the injection of current in PtMn/Ru structures. We believe that the gradual increase of R_{Hall} in PtMn/Ru is associated with the significant temperature rise in these structures, which may lead to electromigration, metastable fragmented domain states [S19], as well as to thermoelectric effects in addition to thermally-assisted Néel vector switching, re-orientation [S11] or nucleation and propagation of domain walls in the structure [S8, S14, S15].

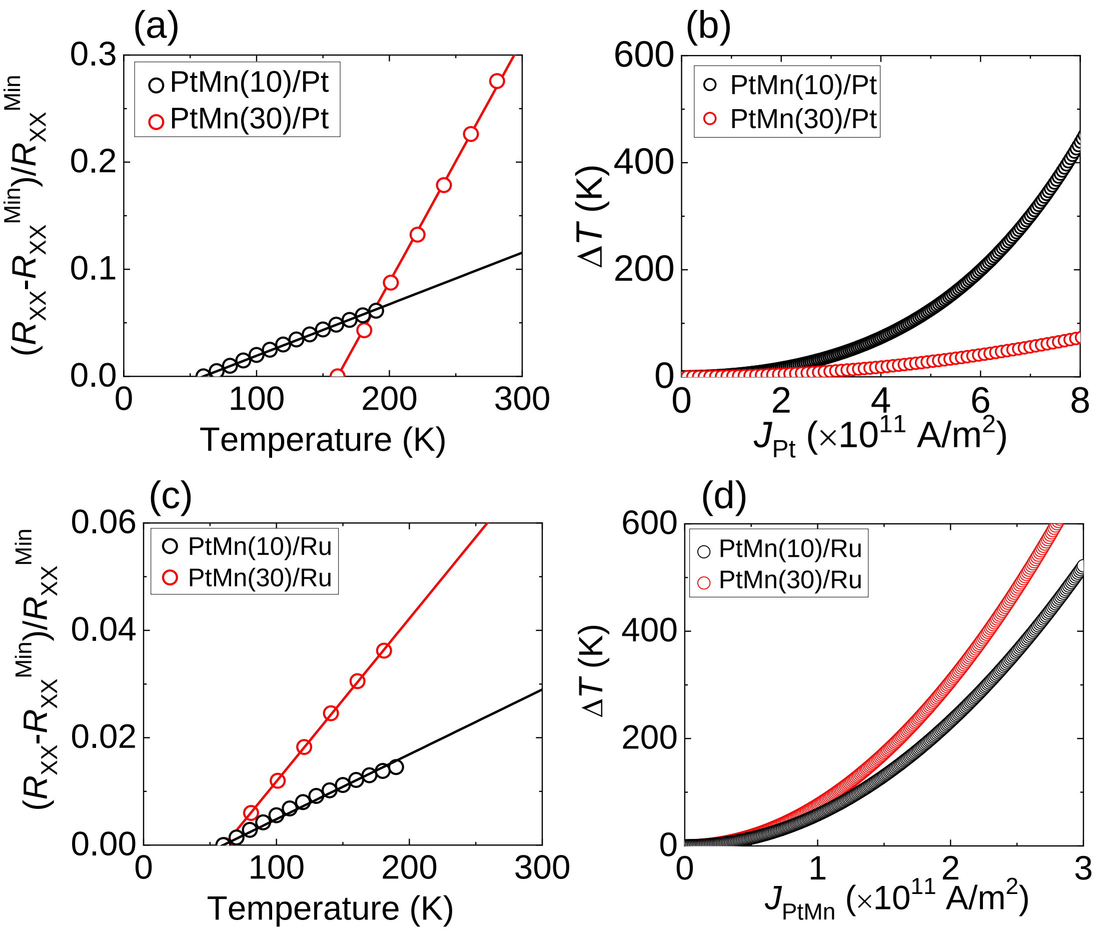

Figure S9: (a) Change in the longitudinal resistance (R_{XX}) of PtMn(10)/Pt and PtMn(30)/Pt structure as a function of temperature. (b) Calculated rise in temperature of the structures versus current density in the Pt (J_{Pt}) layer for PtMn/Pt structures. (c) Change in R_{XX} as a function of temperature for PtMn(10)/Ru and PtMn(30)/Ru structures. (d) Calculated rise in temperature of the structures versus J_{PtMn}.

S11. Optical micrograph of PtMn/Ru structures after current pulse application

To get a qualitative idea concerning the possibility of electrical stress, we take the optical micrograph of a PtMn(10)/Ru device utilized for current-induced switching measurements. The current-induced switching measurements for a similar device from the same wafer are shown in the main text (Fig. 2(b)). We observe darkened regions in the write current paths, while the central cross region remains relatively unaffected (Fig. S10). No significant changes were observed in the read current path. On the contrary, we donot observe any similar changes in PtMn(10)/Pt structures. These changes are indicative of heavy electrical stress owing to the significant current densities in PtMn/Ru structures.

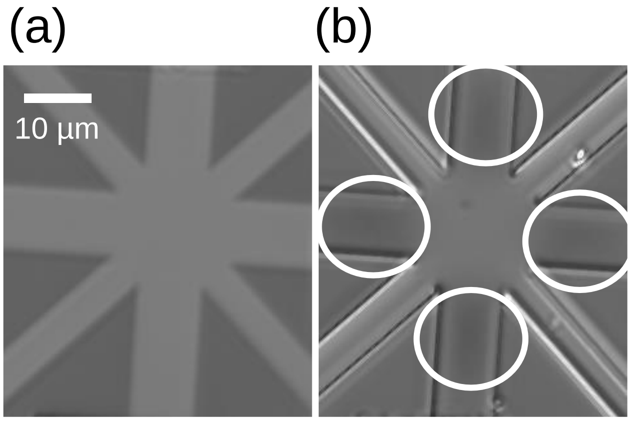

Figure S10: Optical micrograph of PtMn(10)/Ru structure (a) before electrical measurement, and (b) after electrical measurements. The white circled regions in (b) highlights the darkened areas in the device.

References

[S1] W. Mahler, Z. Metallkd. **46**, 282 (1955).

[S2] Ladwig, P. F. *et al.*, Paramagnetic to antiferromagnetic phase transformation in sputter deposited Pt-Mn thin films, J. Appl. Phys. **94**, 979 (2003).

[S3] Peng, T. Y. *et al.*, Study of annealing and exchange bias effects in PtMn based magnetic tunnel junctions, Phys. Stat. Sol. (c) **1**, 3628 (2004).

[S4] Chiang, C. C., Huang, S. Y., Qu, D., Wu, P. H & Chien, C. L., Absence of Evidence of Electrical Switching of the Antiferromagnetic Néel vector, Phys. Rev. Lett. **123**, 227203 (2019).

[S5] Wadley, P. *et al.* Current-polarity-dependent manipulation of antiferromagnetic domains. Nature. Nanotech. **13**, 362 (2018).

[S6] Taylor, J. *et al.*, Magnetic and electrical transport signatures of uncompensated moments in epitaxial thin films of the noncollinear antiferromagnet Mn_3Ir, Appl. Phys. Lett. **115**, 062403 (2019).

[S7] Sakuma A., First-Principles Study of the Non-collinear Magnetic Structures of Disordered Alloys, Journ. Phys. Soc. Jpn. **69**, 3072 (2000).

[S8] Baldarati, L. *et al.* Mechanism of Néel order switching in antiferromagnetic thin films revealed by magnetotransport and imaging techniques, Phys. Rev. Lett. **123**, 177201 (2019).

[S9] Wadley, P. *et al.* Electrical switching of an antiferromagnet. *Science* **351**, 587-590 (2016).

[S10] Bodnar, S. Y. *et al.* Writing and reading of antiferromagnetic Mn_2Au by Néel spin-orbit torques and large anisotropic magnetoresistance. *Nature Commun.* **9**, 348 (2018).

[S11] Meinert, M., Graulich, D. & Mallata-Wagner, T. Electrical Switching of Antiferromagnetic Mn_2Au and the Role of Thermal Activation. *Phys. Rev. Appl.* **9**, 064040 (2018).

[S12] Chen, X. Z. *et al.* Antidamping-Torque-Induced Switching in Biaxial Antiferromagnetic Insulators. *Phys. Rev. Lett.* **120**, 207204 (2018).

[S13] Moriyama, T., Oda, K., Ohkochi, T., Kimata, M. & Ono, T. Spin torque control of antiferromagnetic moments in NiO. *Sci. Rep.* **8**, 14167 (2018).

[S14] Tomasello, R. *et al*. Domain periodicity in an easy-plane antiferromagnet with Dzyaloshinskii-Moriya interaction, arXiv:2004.01944 (2020).

[S15] Sanchez-Tejerina L., Puliafito V., Amiri P. K., Carpentieri M. & Finocchio G., Dynamics of domain-wall motion driven by spin-orbit torques in antiferromagnets, Phys. Rev. B. **101**, 014433 (2020).

[S16] Umetsu, R. Y., Sakuma, A. & Fukamichi, K. Magnetic anisotropy energy of antiferromagnetic L10-type equiatomic Mn alloys. Appl. Phys. Lett. **89**, 052504 (2006).

[S17] Xu, Y., Wang, S. & Xia, K. Spin-Transfer Torques in Antiferromagnetic Metals from First Principles, Phys. Rev. Lett. **100**, 226602 (2008).

[S18] DuttaGupta, S. *et al*. Spin-orbit torques and Dzyaloshinskii-Moriya interaction in PtMn/[Co/Ni] heterostructures, Appl. Phys. Lett. **11**1, 182412 (2017).

[S19] Wörnle, M. S. *et al.* Current-induced fragmentation of antiferromagnetic domains. arXiv:1912.05287v1 (2019).
